# IL-17RA Signaling in Prx1+ Mesenchymal Cells Influences Fracture Healing in Mice

**DOI:** 10.3390/ijms25073751

**Published:** 2024-03-28

**Authors:** Joseph L. Roberts, David Kapfhamer, Varsha Devarapalli, Hicham Drissi

**Affiliations:** 1 Department of Orthopaedics, Emory University, Atlanta, GA 30329, USA; joseph.roberts.3@asu.edu (J.L.R.);; 2 Atlanta VA Health Care System, Decatur, GA 30033, USA; 3 College of Health Solutions, Arizona State University, Phoenix, AZ 85004, USA

**Keywords:** periosteum, secondary bone healing, IL-17A, inflammation, cytokine

## Abstract

Fracture healing is a complex series of events that requires a local inflammatory reaction to initiate the reparative process. This inflammatory reaction is important for stimulating the migration and proliferation of mesenchymal progenitor cells from the periosteum and surrounding tissues to form the cartilaginous and bony calluses. The proinflammatory cytokine interleukin (IL)-17 family has gained attention for its potential regenerative effects; however, the requirement of IL-17 signaling within mesenchymal progenitor cells for normal secondary fracture healing remains unknown. The conditional knockout of IL-17 receptor a (*Il17ra*) in mesenchymal progenitor cells was achieved by crossing *Il17ra*^F/F^ mice with *Prx1-cre* mice to generate *Prx1-cre*; *Il17ra*^F/F^ mice. At 3 months of age, mice underwent experimental unilateral mid-diaphyseal femoral fractures and healing was assessed by micro-computed tomography (µCT) and histomorphometric analyses. The effects of IL-17RA signaling on the osteogenic differentiation of fracture-activated periosteal cells was investigated in vitro. Examination of the intact skeleton revealed that the conditional knockout of *Il17ra* decreased the femoral cortical porosity but did not affect any femoral trabecular microarchitectural indices. After unilateral femoral fractures, *Il17ra* conditional knockout impacted the cartilage and bone composition of the fracture callus that was most evident early in the healing process (day 7 and 14 post-fracture). Furthermore, the in vitro treatment of fracture-activated periosteal cells with IL-17A inhibited osteogenesis. This study suggests that IL-17RA signaling within *Prx1*+ mesenchymal progenitor cells can influence the early stages of endochondral ossification during fracture healing.

## 1. Introduction

Bone fractures are common traumatic injuries that impacted an estimated 178 million people worldwide in 2019 [1]. In most individuals, fractures heal naturally via secondary bone repair that encompasses a complex series of overlapping molecular, cellular, and tissue-level events that lead to the restoration of structural properties of the bone [2]. A robust local inflammatory response is a necessary component of the reparative process that functions to recruit and stimulate the proliferation of mesenchymal progenitor cells to the fracture site. These cells differentiate into chondrocytes and osteoblasts to facilitate the formation of the cartilaginous and bony calluses [3]. The periosteum is the major source of these skeletal stem cells, particularly the chondrocytes that form the soft cartilaginous callus [4]. Several markers that label periosteal mesenchymal progenitor cells during bone regeneration have been identified using lineage-tracing and genetic mouse models. Nearly all the mesenchymal lineage cells (i.e., osteoblasts and chondrocytes) that make up the fracture callus are derived from paired-related homeobox protein 1 (*Prx1*) lineage cells [5,6]. As such, *Prx1*+ cells are considered a primary source of mesenchymal progenitor cells for secondary fracture healing as selective depletion severely impairs bone repair and exacerbates the local inflammatory response [7,8].

Prior work has identified the importance of several inflammatory cytokines and chemokines in stimulating the early stages of fracture healing, including TNF-α [9], IL-1β [10], and IL-6 [11]. More recently, the pleiotropic cytokine IL-17A has gained attention for its potential pro-regenerative effects [12,13,14,15]. Interleukin 17a (IL-17A) is the founding and most thoroughly studied member of the IL-17 cytokine family that is strongly induced in response to infection and injury, including fractures [13,16]. In the context of primary bone repair, IL-17A promoted the osteoblastogenesis of injury-associated mesenchymal cells to facilitate intramembranous ossification [13]. More recently, the infiltration of IL-17A-producing Th17 and γδT cells was reported to be critical for secondary bone repair [12]. In the absence of injury and in the context of homeostasis, IL-17A has been reported to influence the activity of osteoblasts. However, the osteogenic effects of IL-17A are controversial with conflicting reports suggesting either a pro-osteogenic effect [13,17,18,19,20,21], no effect [22], or an anti-osteogenic effect [13,23,24,25,26,27]. IL-17A signals through a receptor complex that consists of subunits of the ubiquitously expressed Interleukin 17 receptor a (IL-17RA) and IL-17RC (either a heterotrimer or heterodimer) that leads to distinct biological effects [28], including cell migration and proliferation [13,17,23], angiogenesis [29,30], inflammation [31], and bone resorption [32]. While prior studies have suggested a role of IL-17 signaling in intramembranous ossification, the specific requirement of IL-17RA signaling in *Prx1*+ mesenchymal progenitor cells for secondary fracture healing remains unknown.

Herein, we describe the effects of the conditional knockout of *Il17ra* in *Prx1*+ mesenchymal cells on secondary bone repair. We hypothesized that IL-17RA signaling would regulate mesenchymal progenitor cell differentiation that would improve bone and cartilage formation during secondary fracture repair. We first assessed the impact of the conditional deletion of *Il17ra* on the healing of non-stabilized femoral fractures in young, skeletally mature mice. Then, we compared the effects of IL-17A on the osteogenic differentiation of fracture-activated periosteal mesenchymal cells in vitro.

## 2. Results

### 2.1. Deletion of Il17ra in Prx1+ Cells Leads to Subtle Improvements in the Intact Skeleton of Female Mice

**Figure 1:**
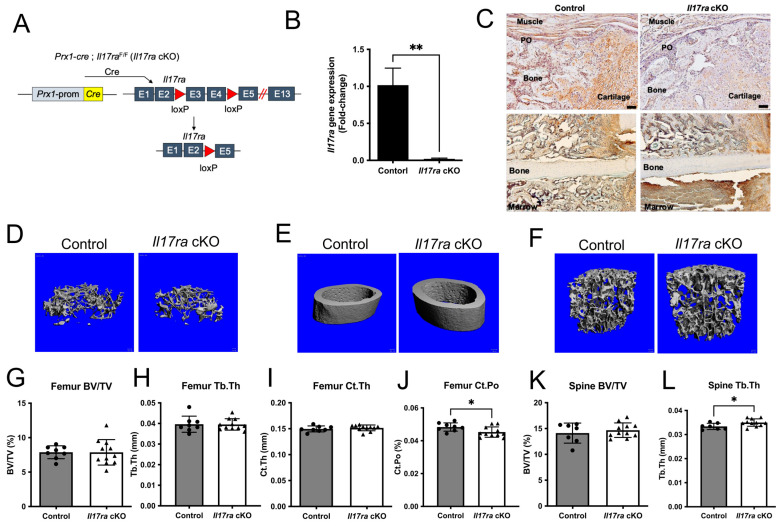
Conditional deletion of *Il17ra* in *Prx1*+ cells leads to subtle changes in bone microarchitecture. (**A**) *Prx1-cre*; *Il17ra*^F/F^ (*Il17ra* cKO) were generated to conditionally delete *Il17ra* in *Prx1*+ mesenchymal cells by deleting exons 3 and 4 which leads to the early termination of translation. (**B**) *Prx1*-*cre* significantly decreased the *Il17ra* gene expression in isolated periosteal cells. (**C**) Immunohistochemistry shows less immunoreactivity of IL-17RA within day 14 fracture calluses and the periosteum of *Il17ra* cKO mice. Scale bar: 50 µm. Representative 3D images of (**D**) femur trabecular bone, (**E**) femur cortical bone, and (**F**) L3 vertebral body trabecular bone. µCT analysis showing (**G**) femur trabecular bone volume fraction (BV/TV), (**H**) femur trabecular thickness (Tb.Th), (**I**) femur cortical thickness (Ct.Th), (**J**) femur cortical porosity (Ct.Po), (**K**) L3 vertebral body trabecular bone volume fraction (BV/TV), and (**L**) L3 vertebral body trabecular thickness (Tb.Th). Abbreviations: PO, periosteum; Prom, promoter; cKO, conditional knockout. Data represent mean ± SD. Student’s unpaired *t*-test, * *p* < 0.05, ** *p* < 0.001.

We sought to determine the influence of IL-17RA signaling in *Prx1*+ mesenchymal cells during fracture healing by deleting *Il17ra* using *Prx1*-*cre* in young 3-month-old mice (Figure 1A). The recombination results in the excision of exons three and four leading to a frame-shift and early termination of translation (Figure 1A). This resulted in a significant decrease in *Il17ra* gene expression in isolated periosteal cells (Figure 1B). Immunohistochemical analyses of IL-17RA within the fracture calluses of day 14 control mice revealed the ubiquitous expression of IL-17RA within cartilage, bone, and periosteum; however, this was decreased within the fracture calluses of *Il17ra* cKO mice (Figure 1C). The conditional deletion of *Il17ra* in *Prx1*+ cells did not influence body weight (Supplementary Figure S1A) or trabecular bone volume fraction (BV/TV), trabecular thickness (Tb.Th), trabecular number (Tb.N), or trabecular separation (Tb.Sp) in the femur (Figure 1D,G,H and Figure S1B,C) or lumbar vertebral body BV/TV, Tb.Sp, and Tb.N (Figure 1F,K, and Supplementary Figure S1E,F). However, Tb.Th was significantly increased in the lumbar vertebral body of *Il17ra* cKO mice (Figure 1L). While the femur cortical thickness (Ct.Th) and cortical area (Figure 1E,I and Supplementary Figure S1D) were unchanged, *Il17ra* cKO did significantly decrease cortical porosity (Ct.Po) (Figure 1J). In male mice, no differences in trabecular bone within the femur and L3 vertebral body or femur cortical bone were observed in *Il17ra* cKO male mice (Supplementary Figure S2). Together, these data demonstrate that *Prx1-cre* efficiently targets *Il17ra* and does not dramatically alter the microarchitecture of the intact skeleton.

### 2.2. Il17ra Conditional Knockout Delays Fracture Healing

**Figure 2:**
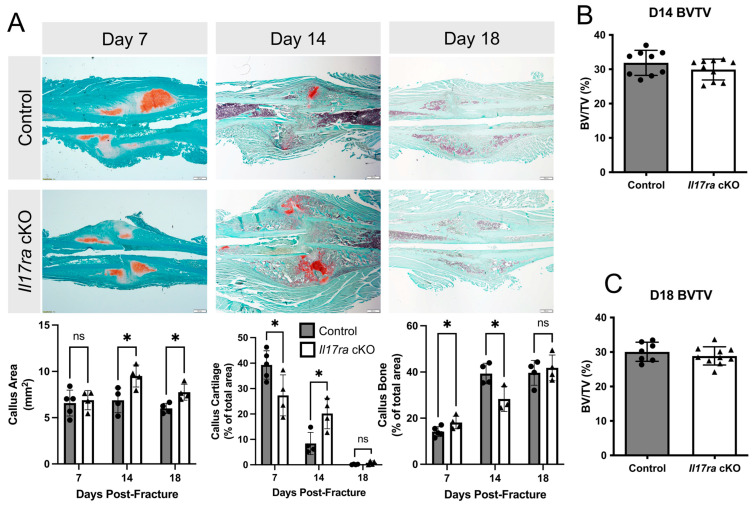
Conditional deletion of *Il17ra* in *Prx1*+ mesenchymal progenitor cells delays healing. (**A**) Representative histological images and static histomorphometric analyses of fracture calluses at day 7, 14, and 18 post-fracture show changes in the composition of the callus across time. Scale bar: 100 µm. µCT volumetric analyses of fracture calluses at (**B**) day 14 and (**C**) day 18 post-fracture. Abbreviations: ns, not significant; BV/TV, bone volume fraction. Data represent mean ± SD. Student’s unpaired *t*-test, * *p* < 0.05.

Our analyses of bone parameters between control and *Il17ra* cKO mice did not reveal many differences in the microarchitecture of intact bone. However, when we challenged the mice with femoral fractures, the *Il17ra* cKO mice exhibited delayed healing. This was indicated by decreased callus cartilage (−30%) and increased callus bone (+27%) in the *Il17ra* cKO mice at day 7 post-fracture (Figure 2A). At day 14 fracture, the calluses were larger (+38%) and contained significantly more cartilage (+140%) and significantly less bone (−28%) in *Il17ra* cKO mice compared to controls (Figure 2A). At day 18 post-fracture, the calluses of *Il17ra* cKO mice were significantly larger (+29%), but no significant differences in callus cartilage or bone were observed compared to the controls (Figure 2A). µCT volumetric analyses did not reveal any differences in callus bone volume fraction (BV/TV) between genotypes at day 14 or day 18 post-fracture (Figure 2B,C). Together, these results indicate that while *Il17ra* cKO in *Prx1*+ cells leads to slight improvements in the intact skeleton, it can lead to a delay the cartilaginous-to-bony callus transition.

### 2.3. IL-17A Inhibits Osteogenic Differentiation of Fracture-Activated Periosteal Cells

**Figure 3:**
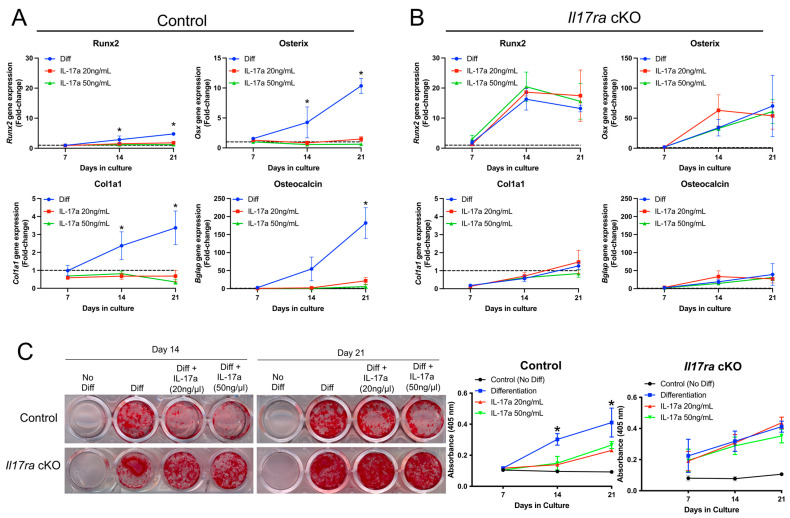
Activation of IL-17RA signaling inhibits osteogenesis. (**A**) IL-17A at 20 and 50 ng/mL inhibited the gene expression of *Runx2*, *Osx*, *Cola1*, and *Bglap* in periosteal cells isolated from control mice. (**B**) IL-17A did not influence expression of *Runx2*, *Osx*, *Cola1*, and *Bglap* in *Il*1*7ra* cKO periosteal cells. (**C**) Alizarin red-S staining and quantification shows less mineralization in IL-17A-treated control periosteal cells at days 14 and 21 of osteogenic differentiation, but there was no effect on mineralization by *Il17ra* cKO cells. Dashed line represented a fold-change of 1. Abbreviations: Diff, osteogenic differentiation; *Osx*, osterix; *Cola1*, collagen type 1; *Bglap,* osteocalcin. Data represent mean ± SD. The two-way ANOVA followed by Tukey’s multiple comparisons, * *p* < 0.05.

The histological data suggested that IL-17RA signaling may contribute to secondary bone formation. We therefore isolated the fracture-activated periosteal cells from control and *Il17ra* cKO mice to determine whether the activation of IL-17RA signaling through its principal ligand, IL-17A, would influence osteogenic differentiation. In periosteal cells isolated from control mice that express IL-17RA (Figure 1B), the addition of IL-17A at a concentration of 20 and 50 ng/mL inhibited osteogenic differentiation as indicated by decreased expression of *Runx2*, Osterix (*Osx*), Collagen type 1 (*Cola1*), and Osteocalcin (*Bglap*) at days 14 and 21 of osteogenic differentiation (Figure 3A). Treating the periosteal cells isolated from *Il17ra* cKO mice with IL-17A did not influence osteogenic gene expression (Figure 3B). Moreover, IL-17A decreased the Alizarin Red-S staining of periosteal cells isolated from control mice at days 14 and 21 of osteogenic differentiation (Figure 3C). This inhibitory effect was not observed *Il17ra* cKO mice (Figure 3C). These data suggest that the activation of IL-17RA signaling by its principal ligand IL-17A can inhibit the osteogenic differentiation of fracture-activated periosteal cells.

### 2.4. IL-17A Increases Fracture-Activated Periosteal Cell Migration In Vitro

**Figure 4:**
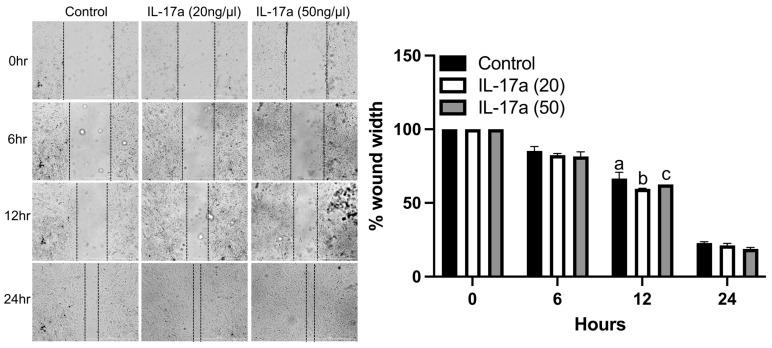
IL-17RA signaling promotes periosteal progenitor cell migration. Wound healing assay shows that IL-17A (20 ng/mL) promoted cell migration at 12 h. (**Left**): representative images of scratched areas marked by black lines. (**Right**): the semi-quantitative analysis of wound closure was determined by measuring the widths of the wounds. Data represent the mean ± SD. Two-way ANOVA followed by Tukey’s multiple comparisons. Values not sharing a common letter differ significantly, *p* < 0.05.

To determine the functional impact of IL-17A on the migration of fracture-activated periosteal cell migration, we performed in vitro wound healing assays using cells isolated from fracture calluses. IL-17A (20 ng/mL) significantly increased the migration of control cells at 12 h compared to the control and 50 ng/mL of IL-17a (Figure 4). There were no significant differences between treatments at 6 or 24 h (Figure 4). These data indicate that the activation of IL-17RA by IL-17A can stimulate the migration of fracture-activated periosteal cells.

## 3. Discussion

This study examined the requirement of IL-17RA signaling in *Prx1*+ mesenchymal cells during secondary bone repair. Our data reveal that the conditional knockout of *Il17ra* leads to a modest improvement in select cortical (femur) and trabecular (L3 vertebral body) bone microstructure indices outside of injury. However, after fracture, *Il17ra* conditional knockout led to an early increase in callus bone (day 7), but delayed the cartilaginous-to-bony callus transition at day 14 post-fracture. We also found that the activation of IL-17RA signaling using its principal ligand IL-17A potently inhibits osteogenic differentiation of fracture-activated periosteal mesenchymal cells in vitro. Taken together, our findings provide new evidence that IL-17RA signaling in *Prx1*+ cells can influence the early stages of secondary bone repair.

We investigated a potential role of IL-17RA during secondary fracture healing and discovered that the absence of the receptor in *Prx1*+ cells increased the callus bone content during early healing (day 7 post-fracture). However, at day 14 post-fracture, we observed more cartilaginous and less bony tissue within the calluses of *Il17ra* cKO mice. These data suggest that the absence of IL17RA signaling in *Prx1*+ mesenchymal cells promotes bone formation during the early stages of secondary fracture healing but delays the cartilaginous-to-bony callus transition that occurs around days 10–14 post-fracture in mice. Several IL-17 family cytokines, including IL-17A, IL-17F, and IL-17E, signal through receptor complexes containing at least one subunit of IL-17RA [28]; however, IL-17A signaling induces a significantly stronger response in downstream gene induction than IL-17F [33]. IL-17F has been shown to enhance osteoblastogenesis by promoting the direct binding between C/EBP-β and the P1 promoter of *Runx2* through the IL-17RA/IL-17RC receptor complex [34,35]. IL-17F was also shown to promote cartilage turnover by increasing the expression of collagenases and decreasing their inhibitors [36,37]. The persistent cartilage within the fracture calluses observed in the *Il17ra* conditional knockouts may partly stem from diminished IL-17F signaling. However, the relative contribution of IL-17A and IL-17F to bone repair during the different stages of fracture healing will need to be delineated in future studies.

There is an acute phase increase in *Il17a* expression that peaks early (day 2) within injured bone and then decreases as the healing progresses [13]. This suggests that IL-17A may be required for the early reactive phase of fracture healing. IL-17A signaling has also been consistently reported to stimulate the migration and proliferation of mesenchymal progenitor cells, which is required for secondary fracture healing [13,17,23,38]. We also observed an increased migration of control fracture-activated periosteal mesenchymal cells in vitro when treated with IL-17A. This may partly explain the delay in fracture healing observed in *Il17ra* cKO mice, which could have been a result of the decreased proliferation and accumulation of mesenchymal progenitor cells at the fracture site. This would culminate in the delayed differentiation of the expanded progenitor cells into cartilage and bone, which we observed in the *Il17ra* cKO mice [38]. However, this needs to be followed-up in future research.

The influence of IL-17A on osteogenesis is controversial, with no clear consensus of its effects on osteogenic differentiation. Some studies classify IL-17A as a pro-osteogenic effector [13,17,18,19,20,21], whereas others report either no effect [22] or the repression of osteogenesis [13,23,24,25,26,27]. In the present study, IL-17A at a concentration of 20 and 50 ng/mL was a potent inhibitor of the osteogenic differentiation and mineralization of periosteum-derived cells. The absence of any effect of IL-17A on osteogenic differentiation in cells lacking IL-17RA and the early increase in callus bone in *Il17ra* cKO mice at day 7 post-fracture further confirms this result. One possible explanation for the inconsistency between studies is the cell type utilized to investigate the osteogenic effect of IL-17A. The studies by Ono et al. demonstrating a pro-osteogenic effect utilized a mixed population of injury-associated mesenchymal cells from the drill hole, periosteum, and surrounding skeletal muscle—whereas those showing an inhibitory effect utilized a more homogenous cell population derived from neonatal mouse calvaria [13]. Importantly, we did not directly determine the impact of *Il17ra* cKO in the presence and absence of IL-17A on the osteogenic differentiation of periosteal cells isolated from intact and fractured bones in our study. This limits our ability to determine whether the *Il17ra* cKO alone or after inflammatory licensing that occurs during healing alters the osteogenic differentiation capacity of mesenchymal cells. Moreover, utilizing *Il17ra* knockdown (siRNA/shRNA) or lenti-cre approaches to deplete *Il17ra* would provide further confirmation of the anti-osteogenic effects of IL-17A in this cell population. These gaps should be investigated in future studies.

We acknowledge several limitations of this study that will direct our future studies in this area. The first is that this study used female mice in the fracture healing phenotyping studies and male mice for in vitro studies. Prior studies reported sex differences in secondary fracture healing as well as in systemic inflammatory responses to fracture [39,40]. Additionally, *Prx1*-*cre* has been reported to lead to partially penetrant germline recombination in female mice, but this was dependent on the particular gene that was floxed [41]. While we did not observe any major sexual dimorphic effects of *Il17ra* cKO on the microarchitecture of the intact skeleton, we cannot exclude the possibility that the response to fracture may differ. Future studies are ultimately needed to determine whether there are any sexual dimorphic effects of *Il17ra* cKO during bone repair. Additionally, we utilized *Il17ra*^F/+^ littermates as our control mice. While we are not aware of any studies reporting skeletal phenotypes in *Prx1*-*cre* or *Il17ra*^F/F^ transgenic mice, we cannot exclude the possibility of off-target effects on bone homeostasis or fracture healing using our current study due to the control we selected. The third is that we did not assess the temporal expression patterns of IL-17RA ligands during secondary fracture healing. IL-17A has been reported to be induced during the early stages of healing, but it is currently unknown whether levels of IL-17A or other IL-17 family cytokines are sustained throughout the healing cascade. Finally, we did not assess the impact of IL-17A on the migration of cells isolated from *I17ra* cKO mice, which should be addressed in future studies.

## 4. Materials and Methods

### 4.1. Animal Husbandry

The *Prx1*-*cre* mice were from Jackson Labs (Bar Harbor, MI, USA #005584). The *Il17ra*^F/F^ mice were kindly provided by Dr. Michael Karin (University of California, San Diego) and have previously been described [42]. *Prx1*-*cre* mice were bred with the *Il17ra*^F/F^ mice to generate *Prx1*-*cre*;Il17ra^F/+^ mice, which were then crossed with *Il17ra*^F/F^ mice to generate experimental *Prx1*-*cre*;*Ill17ra*^F/F^ (*Il17ra* cKO) mice. Conditional knockout mice were compared to littermate *Il17ra*^F/+^ controls. Mice were group housed with ab libitum access to autoclaved food (Envigo, Madison, WI, USA #2018S) and water (0.1 micron-filtered). All mice were housed at the Atlanta Veterans Affairs Medical Center (VAMC) vivarium in specific pathogen-free cages and controlled conditions (temperature, 21–24 °C; humidity, 40–70%; light/dark cycle, 12/12 h). Mice were maintained in accordance with an applicable state and federal guidelines and all experimental procedures were approved by the Atlanta VAMC Institutional Animal Care and Use Committee.

### 4.2. Fracture Model

Femoral fractures were generated using the Einhorn method as we previously described [43,44,45,46]. Three-month-old mice were anesthetized with isoflurane inhalation, given analgesics (Buprenorphine SR, Fidelis, North Brunswick, NJ, USA) subcutaneously, and the left hind limb shaved and sterilized with chlorohexidine and isopropyl alcohol. The articular surface of the femoral intercondylar notch was then perforated with a 25-gauge needle through the skin, followed by the insertion of a precut stainless steel 316LVM wire (diameter 0.15 inch) into the medullary canal with the use of a retrograde approach. A transverse mid-diaphyseal fracture was then created using three-point bending via a blunt guillotine device. The fractured limbs were radiographically examined by digital X-ray (Bruker, Billerica, MA, USA) immediately post-fracture to confirm the fracture location and pin placement. Animals with comminuted, distal, or proximal fractures were excluded from phenotyping analyses. Mice were allowed to fully weight-bear without any restrictions on activity after recovery from anesthesia. At either 5, 7, 14, or 18 days post-fracture, mice were euthanized by CO_2_ asphyxiation followed by cervical dislocation.

### 4.3. Micro-Computed Tomography

Micro-computed tomography (µCT) was performed on the fractured femur to determine callus bone. µCT was also performed on the unfractured contralateral femur and 3rd lumbar (L3) vertebrae ex vivo to assess the trabecular and cortical bone microarchitecture using a µCT40 scanner (Scanco Medical AG, Brüttisellen, Switzerland) that was calibrated weekly using a factory-supplied phantom. Bones were first fixed for 1 week in 10% neutral buffered formalin at 4 °C followed by scanning in PBS medium. For fracture callus analyses, fracture calluses were manually segmented to exclude existing cortical bone and any bone fragments at the center of the fracture callus as previously described [44]. A Gaussian filter (sigma = 0.8, support = 1) was applied to reduce the noise and the following measures of the callus structure and composition were quantified for each fracture callus–bone volume fraction (BV/TV).

Microarchitecture of the unfractured contralateral femurs were analyzed from 99 tomographic slices taken from the distal femoral metaphysis starting 0.5 mm proximal from distal growth plate. Trabecular bone was manually segmented from the cortical shell at a voxel size of 6 µm (70 kVp and 114 mA, and with 200 ms integration time). Cortical bone was quantified at the femoral mid-diaphysis from 104 tomographic slices. Projection images were reconstructed using the auto-contour function for vertebral body trabecular bone between the cranial and caudal growth plates from approximately 350 tomographic slices. The following 3D indices in the defined ROI were analyzed: relative bone volume over total volume (BV/TV, %), trabecular thickness (Tb.Th, mm), trabecular number (Tb.N, 1/mm), trabecular separation (Tb.Sp, mm), cortical porosity (Ct.Po, %), cortical area (Ct.Ar, mm^2^), and cortical thickness (Ct.Th, mm). All indices and units were standardized according to the published guidelines [47].

### 4.4. Histology, Immunohistochemistry, and Static Histomorphometry

After µCT scanning, fractured femurs were decalcified in 14% EDTA (pH 7.2) for ≥2 weeks before embedding in paraffin. Five-micrometer-thick sections were obtained and stained with Safranin O/Fast green. The percent of cartilage and bone within the fracture callus was quantified using Osteomeasure (Osteometrics, Decatur, GA, USA) by normalizing the amount of each tissue type to the size of the callus. For immunohistochemical analyses, midsagittal histological sections (5 µm thick) were deparaffinized and rehydrated through a graded alcohol series. Sections were then treated with 3% hydrogen peroxide to quench endogenous peroxidase activity. Antigen unmasking was conducted by incubating sections in citrate buffer at 55 °C overnight in a water bath. Sections were blocked with 10% normal Goat serum for 1 h then incubated overnight with Rabbit Anti-IL-17RA antibody (1:100 in 1.5% normal Goat serum; Abcam, Boston, MA, USA #ab218249) in a humidified chamber placed at 4 °C. After the overnight incubation, sections were incubated with secondary antibody (SignalStain Boost IHC Detection Reagent HRP, Rabbit, Cell Signaling, Danvers, MA, USA #8114) for 30 min. Sections were stained with 3,3′-diaminobenzidine tetra-hydrochloride (SignalStain DAB Substrate Kit, Cell Signaling, Danvers, MA, USA #8059s) and counter-stained with hematoxylin.

### 4.5. Periosteal Cell Isolation

Periosteal cells were isolated from day 5 fractured femora as previously described [48]. Briefly, muscle was carefully removed without disturbing the periosteal layer; then, the bone marrow was removed by first removing the intramedullary K-wire and then cutting the epiphysis and flushing with alpha-MEM. The periosteum was scraped from the diaphyseal cortex and callus before being digested in HBSS containing 0.125% trypsin (no EDTA) (Life Technologies, Carlsbad, CA, USA), 1 mg/mL Collagenase A (Sigma, St. Louis, MO, USA; Cat# 10103586001), and 1 mg/mL hyaluronidase (Sigma, St. Louis, MO, USA; Cat# H3506) for 1 h at 37 °C with agitation. The digested tissue was then strained through a 70 µm filter, centrifuged, and resuspended in alpha-MEM supplemented with 20% FBS (Atlanta Biologicals, Flowery Branch, GA, USA) and 1% penicillin–streptomycin (Life Technologies, Carlsbad, CA, USA). Periosteal cells were expanded at 37 °C in hypoxic conditions (5% O_2_ and 5% CO_2_) until confluence (approximately 7–8 days) before use in osteogenesis experiments.

### 4.6. Periosteal Cell Osteogenic Differentiation

Isolated periosteal cells were plated at 3 *×* 10^4^ cells/well of a 24-well plate and cultured in alpha-MEM supplemented with 10% FBS (no differentiation control), or in a differentiation medium containing 50 µg/mL ascorbic acid (Sigma, St. Louis, MO, USA) and 8 mM 2-glycerol phosphate (Sigma, St. Louis, MO, USA). To test the effects of IL-17a on fractured activated periosteal cells, murine IL-17A (Peprotech, Cranbury, NJ, USA; #210-17) was added to a differentiation medium at a final concentration of 20 and 50 ng/mL. Cells with cultured at 37 °C in normoxic (20% O_2_ and 5% CO_2_) conditions. Medium was refreshed every 3 days and cells were harvested for RNA as described below and stained with Alizarin Red-S (40 mM, pH 4.2) at 7, 14, and 21 days. Briefly, cells were fixed in 10% neutral buffered formalin for 15 min before being stained with Alizarin Red-S for 20 min at room temperature. Dye was extracted using 10% acetic acid and absorbance quantified using a SpectraMax M2 plate reader (Molecular Devices, San Jose, CA, USA).

### 4.7. Gene Expression

Isolated periosteal cells (described above) from the control and conditional knockout mice were cultured under basal conditions, osteogenic conditions, or osteogenic conditions containing IL-17A (20 and 50 ng/mL) for 7, 14, and 21 days. Total RNA was isolated from cells using TRIzol (Invitrogen, Waltham, MA, USA) according to the manufacturer’s instructions. First-strand cDNA was synthesized with oligo(dT) and random primers using qScript cDNA SuperMix (Quantabio, Beverly, MA, USA). All qRT-PCR were performed on an Analytik Jena qTower3 G Real-Time PCR Detection System using Applied Biosystems (Waltham, MA, USA) PowerUp SYBR Green master mix. Amplicon authenticity was confirmed by the melt curve analysis. Primer sequences are provided in Supplementary Table S1 and β-actin was used as the normalization control. The data were analyzed for fold-change using the ∆∆CT method.

### 4.8. Wound Healing Assay

Periosteal cells, isolated from day 5 fracture calluses, were cultured to confluent monolayers in alpha-MEM supplemented with 10% FBS and 1× penicillin–streptomycin. Straight wounds were made by using a 200 µL pipette tip. After washing with 1× PBS to remove cell debris, wounded monolayers were incubated in alpha-MEM supplemented with 10% FBS supplemented with 20 or 50 ng/mL IL-17A (Peprotech, Cranbury, NJ, USA). The wound gaps were imaged at 0, 6, 12, and 24 h using a Lionheart LX microscope (BioTek, Winooski, VT, USA), and the area of cell-free wounds was measured using ImageJ (version 1.53t).

### 4.9. Statistics

Results are shown as the mean ± SD. Statistical significance was determined by unpaired two-tailed Student’s *t*-test or two-way ANOVA followed by Tukey’s multiple comparisons test using GraphPad Prism software (version 8.3.0). All statistical tests were performed at the 5% significance level.

## 5. Conclusions

In summary, this study established that, in the absence of injury, *Il17ra* cKO in *Prx1*+ mesenchymal cells leads to a decrease in femoral cortical porosity in female mice. However, upon fracture, *Il17ra* cKO led to an increased callus bone content early during the post-fracture period but delayed the cartilaginous-to-bony callus transition. Activating IL-17RA signaling with IL-17A also inhibited the osteogenic differentiation of fracture activated periosteal cells. This study sheds new light into the role of IL-17RA signaling during bone repair.

## Data Availability

The datasets generated and analyzed for the current study are available from the corresponding author upon reasonable request.

## Supplementary Materials

The following supporting information can be downloaded at: https://www.mdpi.com/article/10.3390/ijms25073751/s1.
