# Supplementary material for: IL-17RA Signaling in Prx1+ Mesenchymal Cells Influences Fracture Healing in Mice
Source: Int J Mol Sci. 2024 Mar 28;25(7):3751. doi: 10.3390/ijms25073751 (PMC11011315; doi:10.3390/ijms25073751)
Supplement: Supplementary file 1 [file ijms-25-03751-s001.zip › Supplementary Figures S1 and S2.pdf]

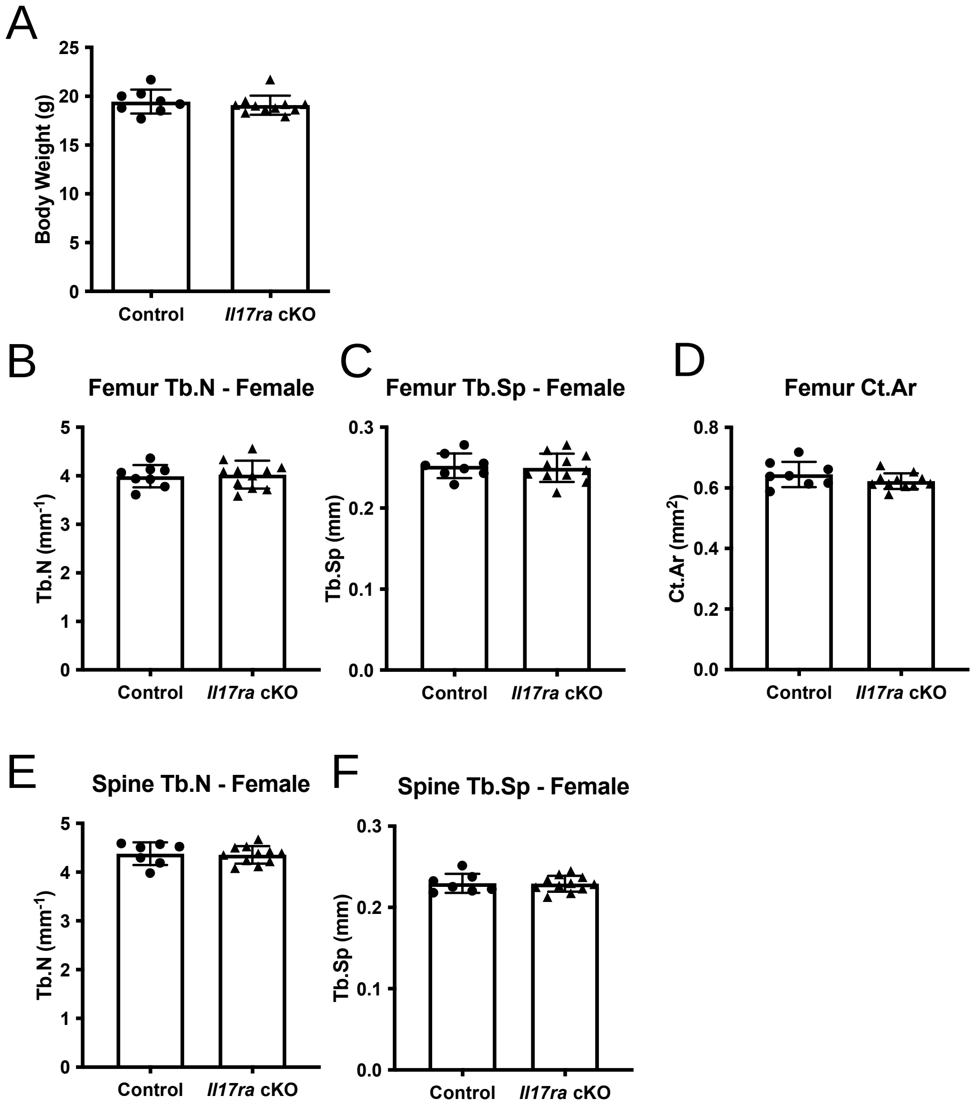

Supplementary Figure S1. Body weight and μCT indices from female mice.

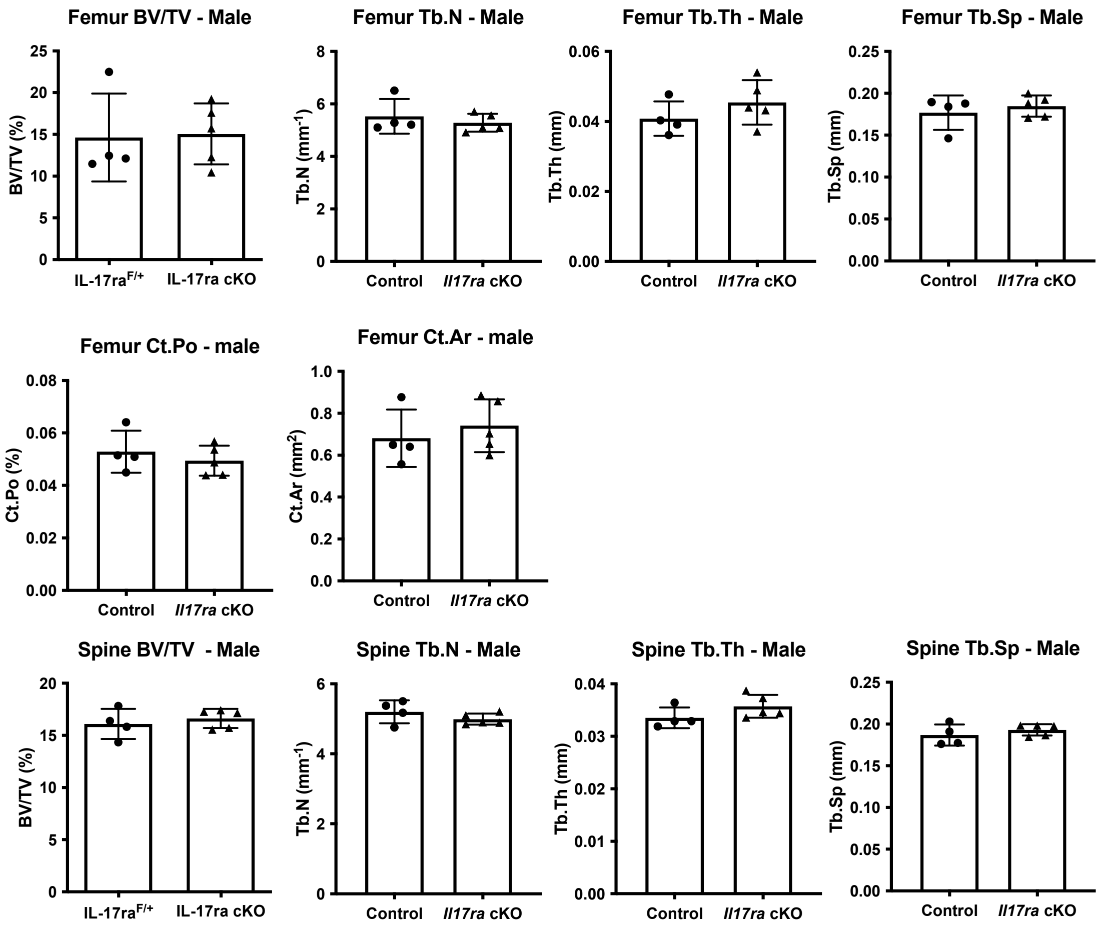

Supplementary Figure S2. μCT indices from male mice.
