# Supplementary material for: IL-17RA Signaling in Prx1+ Mesenchymal Cells Influences Fracture Healing in Mice
Source: Int J Mol Sci. 2024 Mar 28;25(7):3751. doi: 10.3390/ijms25073751 (PMC11011315; doi:10.3390/ijms25073751)
Supplement: Supplementary file 1 [file ijms-25-03751-s001.zip › Supplementary Table S1.pdf]

Supplementary Table S1. Primer sequences.

Gene	Sequence (5'→3')	Product Size (bp)
Runx2	GACATCCCCATCCATCCACT<br>TGAGAGAGGAAGGCCAGAGG	140
Osx	ACTGGCTAGGTGGTGGTCAG<br>GGTAGGGAGCTGGGTTAAGG	135
Cola1	TGTCCCAACCCCCAAAGAC<br>CCCTCGACTCCTACATCTTCTGA	92
Bglap	TGCTTGTGACGAGCTATCAG<br>GAGGACAGGGAGGATCAAGT	149
IL-17ra	AGTGTTTCCTCTACCCAGCAC<br>GAAAACCGCCACCGCTTAC	194
β-actin	AGATGTGGATCAGCAAGCAG<br>GCGCAAGTTAGGTTTTGTCA	125
